# mRNA and microRNA analysis reveals modulation of biochemical pathways related to addiction in the ventral tegmental area of methamphetamine self-administering rats

**DOI:** 10.1186/s12868-015-0186-y

**Published:** 2015-07-19

**Authors:** P J Bosch, M C Benton, D Macartney-Coxson, B M Kivell

**Affiliations:** Centre for Biodiscovery, School of Biological Sciences, Victoria University of Wellington, Kelburn Parade, PO Box 600, Wellington, 6140 New Zealand; Institute of Environmental Science and Research, Wellington, New Zealand; Genomics Research Centre, Institute of Health and Biomedical Innovation, Queensland University of Technology, Brisbane, Australia

**Keywords:** Brain, Genetics, Methamphetamine, Self-administration, microRNA

## Abstract

**Background:**

Methamphetamine is a highly addictive central nervous system stimulant with increasing levels of abuse worldwide. Alterations to mRNA and miRNA expression within the mesolimbic system can affect addiction-like behaviors and thus play a role in the development of drug addiction. While many studies have investigated the effects of high-dose methamphetamine, and identified neurotoxic effects, few have looked at the role that persistent changes in gene regulation play following methamphetamine self-administration. Therefore, the aim of this study was to identify RNA changes in the ventral tegmental area following methamphetamine self-administration. We performed microarray analyses on RNA extracted from the ventral tegmental area of Sprague–Dawley rats following methamphetamine self-administration training (2 h/day) and 14 days of abstinence.

**Results:**

We identified 78 miRNA and 150 mRNA transcripts that were differentially expressed (fdr adjusted p < 0.05, absolute log2 fold change >0.5); these included genes not previously associated with addiction (miR-125a-5p, miR-145 and *Foxa1*), loci encoding receptors related to drug addiction behaviors and genes with previously recognized roles in addiction such as miR-124, miR-181a, *DAT* and *Ret.*

**Conclusion:**

This study provides insight into the effects of methamphetamine on RNA expression in a key brain region associated with addiction, highlighting the possibility that persistent changes in the expression of genes with both known and previously unknown roles in addiction occur.

**Electronic supplementary material:**

The online version of this article (doi:10.1186/s12868-015-0186-y) contains supplementary material, which is available to authorized users.

## Background

Methamphetamine is a highly addictive psychostimulant reported to be the second most highly abused illegal drug in the world [1]. Intoxication causes euphoria and hyperactivity, as well as depression, anxiety and psychosis [2, 3]. Extensive gene expression changes following high levels of methamphetamine have been observed in the brain, causing dopaminergic terminal degeneration in many rodent models [2, 4].

Pre-clinical research using experimenter-administered (non-contingent) exposure to methamphetamine has shown extensive gene expression changes in various brain regions [5–7]. Acute experimenter-administered methamphetamine increases the expression of a number of immediate early genes, including those encoding transcription factors, *c*-*fos*, *arc*, *NFκB*, *preprodynorphin*, *fra2*, *Egr1*-*3*, *Nr4a1* and *Nr4a3* in the striatum [8, 9]. Chronic methamphetamine exposure has been shown to alter genes involved in GTPase signaling, apoptosis, and cell cycle control in the striatum, in addition to the well-established addiction associated genes *fos*, *arc* and *prodynorphin* [10]. Investigation of methamphetamine self-administration has shown that contingent exposure elicits different neurobiological consequences to non-contingent exposure [11] and is a method with greater face validity compared with experimenter-administered models [12]. Recently, Krasnova et al. performed an extensive transcription survey in the dorsal striatum following methamphetamine self-administration for 15 h/day, in which gene expression changes persisted for up to 1 month of abstinence [13]. Previously, short access methamphetamine self-administration has been shown to transiently reduce dopamine D_2_ receptor expression in the ventral tegmental area (VTA) using in vitro quantitative autoradiography following 24 h abstinence [11], and to elicit a sensitized dopamine and glutamate response in the nucleus accumbens (NAc) following a 2 mg/kg challenge injection of methamphetamine [14]. This suggests that short-access models can be used to study neuroadaptations in the absence of dopaminergic neurotoxicity.

Micro-RNA (miRNA) are ~22 nucleotide RNA molecules that act to regulate the expression of mRNA, by binding to their 3′ untranslated region (3′UTR); this leads to translational inhibition or transcriptional repression [15]. In the brain, enriched miRNA appear to target genes with increased and/or tissue-specific expression, and are thought to act to subtly modulate gene expression networks regulated by many factors including transcriptional activators [16]. MiRNAs have a significant role in modulating the effects of drugs of abuse including cocaine, alcohol, nicotine, and opioids within brain reward circuitry [17]. They are involved in the development of synaptic connections and plasticity, direct dendrite formation in neurons [18], and have an important role in the development of addiction-related behaviors [19]. The VTA contains dopaminergic cell soma and innervates other brain regions, including the NAc and prefrontal cortex [20], which are the primary regions of methamphetamine’s pharmacological effects [21]. VTA neurons have a role in reward and drug reinforcement [22] and drug-seeking [23] and as such this is an important brain region to study for persistent changes following drug administration. Therefore, given the importance of the VTA in addiction, the potential relevance of the rat drug self-administration model to human drug-taking patterns, the importance of gene expression changes in addiction and the dearth of such RNA data for the VTA, we sought to study miRNA and mRNA expression in the VTA using a methamphetamine self-administration model followed by 14 days of abstinence.

## Results

### Methamphetamine self-administration

**Figure 1:**
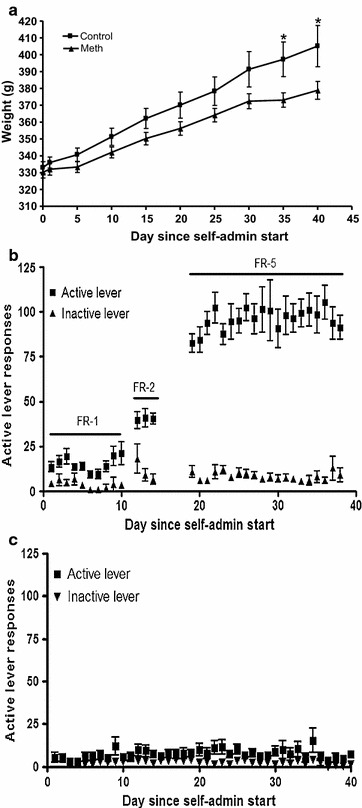
**a** Rat weight during the course of the self-admin study. Methamphetamine self-administration rats (n = 11) gained less weight than controls (n = 11) for the duration of the study; the weights were significantly different at day 35 and day 40 (p < 0.05, Student’s t-test). **b** Number of active and inactive lever responses over the course of the study for methamphetamine self-administration rats, and **c** control rats lever responses.

Rats trained for methamphetamine self-administration showed preference for the active lever over the inactive lever on FR-1, FR-2 and FR-5 schedules of reinforcement, as expected from previous studies [24]. The rats that self-administered methamphetamine gained weight at a slower rate than the control rats during the course of the study (Figure 1a).

Two-way ANOVA between active and inactive lever responses of the methamphetamine self-administration rats revealed a significant effect of lever [F(1,20) = 115.1, p < 0.0001] over the 20 FR-5 sessions. There was no significant effect of time [F(19,380) = 0.4254, p = 0.9848] or interaction [F(19,380) = 0.9515, p = 0.5189].

Body weight was significantly different between the control and methamphetamine self-administration groups on days 35 and 40 (p < 0.05, Student’s *t* test). Rats did not display escalation of drug intake during the course of the study; the average total methamphetamine intake across the whole study was 43.9 ± 5.4 mg/kg (range 26.1–58.2 mg/kg total intake, Figure 1b). The control group did not show a preference for the active lever over the inactive lever and received an average of 0.9 mL/day of the heparinized saline solution, compared with 2.2 mL/day for the methamphetamine self-administering rats (Figure 1c).

### mRNA expression changes following methamphetamine self-administration in rats

**Figure 2:**
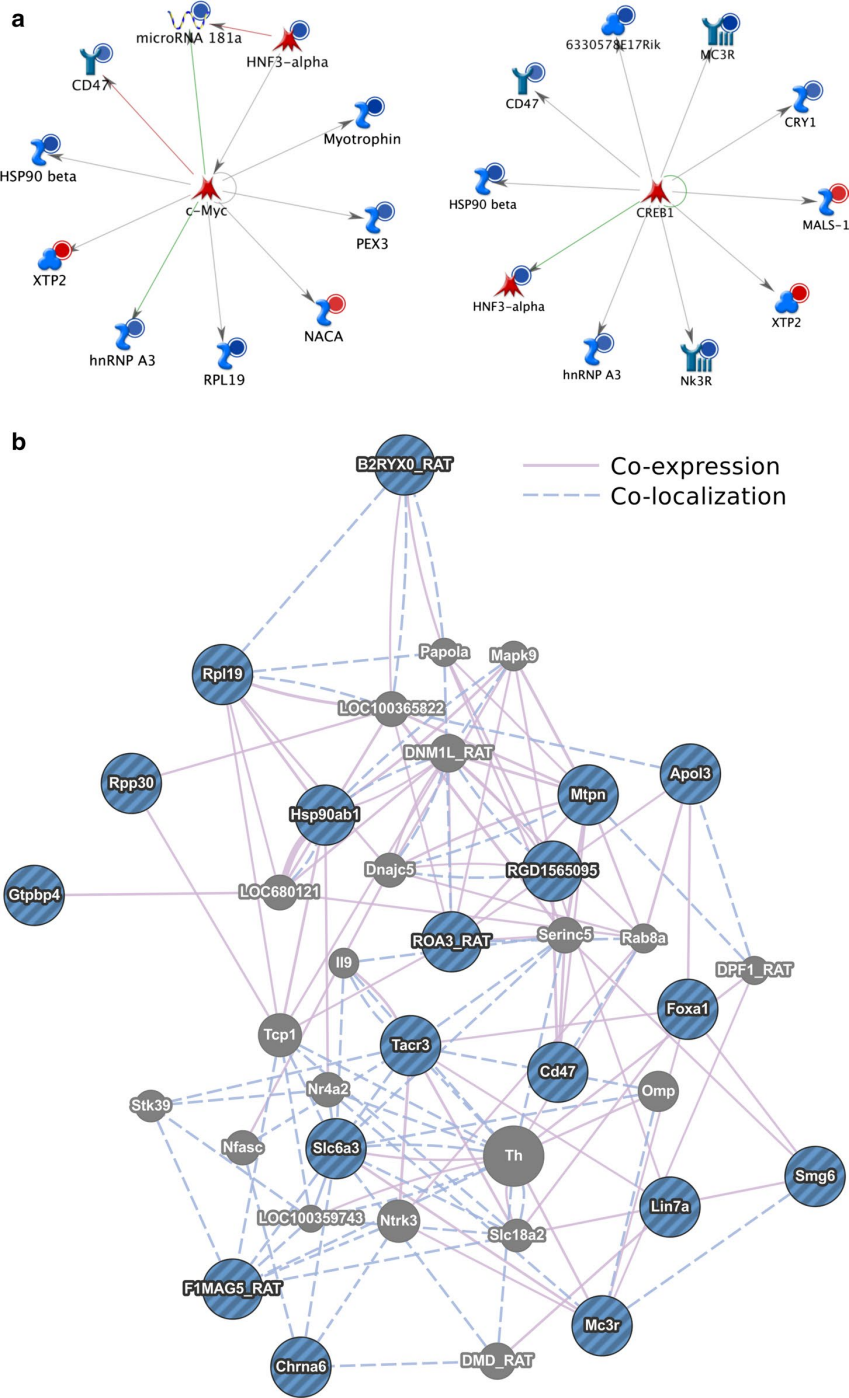
**a** Significant enrichment of genes regulated by transcription factors, c-Myc and CREB. Key: *blue circles* upregulation; *red circles* downregulation. Significant values for both enrichments were p-value = 1.62e−32, zScore 123.52 and gScore 123.52. **b** Enrichment of 18 of the 48 differentially-expressed mRNA transcripts which are either co-expressed or co-localized. Key: Myotrophin (Mtpn); B2RYX0_RAT, Naca; ROA3_RAT, Hnrnpa3; RGD1565095, Coa5; F1MAG5_RAT, Ret.

Unsupervised hierarchical clustering of the mRNA expression data grouped the samples by treatment (Additional file 1: Figure S1). Differential expression of 150 transcripts was observed at an adjusted p < 0.05 (BH) and an absolute log2 fold change >0.5. Of these, 48 mapped to annotated genes, with 17 showing downregulation and 31 upregulation (Table 1). Pathways analysis of the 48 annotated mRNA revealed significant enrichment for three processes important in addiction: regulation of dopamine metabolic process (adj p = 2.02 × 10^−2^), regulation of biological quality (adj p = 2.02 × 10^−2^) and genes integral to the plasma membrane (adj p = 9.90 × 10^−3^). We observed upregulation of the precursor transcript miR-181a-2 on the Exon array (fold change (log2) = −0.69, adj p = 0.00085). Furthermore, genes targeted by two transcription factors, c-Myc and cAMP response element binding protein (CREB) were highly enriched within the mRNA dataset (p < 1.7 × 10^−32^) (Figure 2a). In addition, pathways enrichment of protein–protein interactions revealed a core group of 18 of the 48 differentially-expressed mRNA that show evidence-based co-expression and/or co-localization. These included cell surface proteins DAT (Slc6a3), Ret (F1MAG5_RAT), Tachykinin receptor (Tacr3), Melanocortin receptor (Mc3r) Nicotinic cholinergic receptor (Chrna6) and Hnrnpa3 (ROA3_RAT), as well as the transcription factor, Foxa1 and scaffold protein Lin7a (Figure 2b).

**Table 1:** Differential expression of mRNA from methamphetamine self-administration rats

Gene name	Fold change (log2)	p-value	miRNA target^a^
Upregulated			
Endosomes			
Slc9a6	−0.51	0.0025	miR-181a/d, 16, 195, 124
Mtpn	−1.00	0.0028	miR-124, 9, 181, 140, 143, 26a, let-7
Myo18b	−0.53	0.0049	
Cell signaling			
Ankh	−0.56	9.30E−05	miR-9
Ppm1h	−0.56	0.00087	miR-125a-5p/b-5p, 351
Gpr64	−0.60	0.0012	miR-23a/b
Cd47	−0.52	0.0016	miR-181d, 9
Neurite growth/extension			
Ntn1	−0.75	0.0010	let-7d/e, miR-27a/b, 20a, 106b
LOC691277	−0.52	0.0040	
Neuroprotection			
Pex3	−0.64	0.00052	miR-30b-5p/c/d
Coa5/6330578E17Rik	−0.87	0.00024	
Ret	−0.88	0.0023	miR-23a/b, 128, 27a/b, 125a-5p/b-5p
Hsp90ab1	−0.82	0.0030	
RNA processing			
Hnrnpa3	−0.62	0.00034	miR-221, 222, 206
Rpp30	−0.55	0.0022	
Rpl19	−0.94	0.0037	
LOC100359671	−0.55	0.0049	
Membrane transporters/receptors			
Olr527	−1.05	0.00040	
Slc6a3	−2.45	0.0010	
RGD1561777	−0.82	0.0014	
Mc3r	−1.03	0.0030	
Tacr3/Nk3R	−0.83	0.0031	
Chrna6	−1.76	0.0045	
Slc47a2	−0.51	0.0047	
Transcriptional regulation			
Foxa1/HNF3-alpha	−0.78	0.00054	miR-106b, 194, 30b-5p/c, 20a
Mir181a-2	−0.69	0.00085	
LOC690309	−0.78	0.0012	miR-26a, 29a/b/c, 222, 383
Pfdn1	−0.67	0.0014	
Smg6	−0.51	0.0029	
Other			
Cry1	−0.58	0.0010	
Samd9 l	−0.69	0.0038	
Downregulated			
Endosomes			
Dnah3	0.63	0.00021	
Lin7a/MALS-1	0.64	0.00043	
LOC494539	0.51	0.0029	miR-125a-5p
Ifitm7	0.72	0.0039	
Cell signaling			
Dkk3	0.66	0.0011	let-7
Gtpbp4	0.78	0.0012	
Membrane transporters/receptors			
Olr625	0.64	0.00048	
Vom1r2	0.69	0.0018	
Vom1r26	0.93	0.0022	
Olr1373	0.63	0.0033	
Transcriptional regulation			
Naca	0.57	0.00066	
Other			
LOC690000	0.53	0.00096	
LOC691519	0.50	0.0018	
LOC691988	0.84	0.0029	
Senp17	1.34	0.0029	
Apol3	1.14	0.0031	
XTP2	0.90	0.0046	

Canonical pathway^b^	mRNA	p-value	RER
Ribosome biogenesis in eukaryotes	Rpp30, Gtpbp4	0.0024	27.45
Neuroactive ligand-receptor interaction	Tacr3, Chrna6, Mc3r	0.0019	12.27
RNA transport	Rpp30, Senp17	0.0081	14.78
Pathways in cancer	Hsp90ab1, Ret	0.0313	7.23

*RER* ratio of enrichment.

^a^Listed are miRNA transcripts significantly differentially expressed in the microarray experiment.

^b^Determined using WebGestalt.

We also interrogated for potential mRNA:mRNA correlations between the significantly differentially-expressed mRNA transcripts (Pearsons). Distinct differences were observed between mRNA showing strong correlations in their expression levels (R > 0.7) in drug naïve compared to methamphetamine self-administration samples (Additional file 2: Figure S2).

### microRNA expression changes following methamphetamine self-administration in rats

Unsupervised hierarchical clustering of the miRNA expression data grouped the samples principally by drug administration (Additional file 3: Figure S3). Differential expression of 78 precursor and mature miRNA was observed at an adjusted p < 0.05 (BH) and an absolute log2 fold change >0.5 with the majority of these miRNA (n = 71) downregulated in methamphetamine self-administration rats compared to drug naïve controls (Table 2, Additional file 4: Table S1 for full list).

**Table 2:** Significantly differentially expressed miRNA with methamphetamine self-administration

Full I.D.	I.D.	Fold change (log2)	Adj p-value	B
Downregulated				
rno-miR-27a_st	Mir27a	2.95	0.041	−2.7
rno-miR-378_st	Mir378	2.5	0.031	−1.94
rno-miR-129_st	Mir129	2.48	0.025	1.42
rno-miR-29c_st	Mir29c	2.39	0.025	1.3
rno-miR-128_st	Mir128	2.36	0.04	−2.64
rno-miR-9*_st	Mir9*	2.36	0.049	−3.03
rno-miR-146a_st	Mir146a	2.35	0.026	−0.92
rno-miR-192_st	Mir192	2.32	0.035	−2.31
rno-miR-30d_st	Mir30d	2.31	0.028	−1.43
rno-miR-106b_st	Mir106b	2.31	0.049	−3.06
Upregulated				
rno-miR-741-3p_st	Mir741-3p	−0.51	0.025	1.26
rno-miR-3570_st	Mir3570	−0.53	0.025	−0.55
rno-miR-369-3p_st	Mir369-3p	−0.60	0.025	−0.52
rno-miR-145*_st	Mir145*	−0.50	0.029	−1.81
hp_rno-mir-216b_st	Mir216b	−0.60	0.033	−2.09
hp_rno-mir-17-1_st	Mir17-1	−0.57	0.032	−2.03
hp_rno-mir-181b-1_st	Mir181b-1	−0.54	0.033	−2.13

Top 10 downregulated miRNA shown, for full table, see Additional file 4: Table S1.

### Overlap of miRNA and mRNA

mRNA expression/stability can be regulated by miRNAs. Therefore we interrogated the 3′UTR of differentially-expressed mRNA for putative miRNA binding sites and investigated whether any of these miRNA were differentially expressed in our analyses. This revealed 12 transcripts which were up-regulated in comparison to a downregulation of their respective putative target miRNA (Table 1). In addition, we performed miRNA enrichment analyses for the 48 differentially expressed mRNA (Table 3). Of these, two (miR-9, adj p = 0.0228 and miR-145, adj p = 0.0298) that were identified as significantly enriched, were also significantly differentially-expressed in the miRNA microarray results.

**Table 3:** Significantly enriched miRNA using the mRNA dataset (WebGestalt)

Micro RNA	Pathways enrichment	
	Raw p-value	Adjusted p-value (Bonferroni)
miR-509	0.0008	0.0096
miR-141, miR-200a	0.0023	0.0138
miR-149	0.0069	0.0228
*miR-9*	*0.0076*	*0.0228*
miR-518a-2	0.0122	0.0293
*miR-145*	*0.0149*	*0.0298*
miR-182	0.0304	0.0521

Italics indicate miRNA that were identified as differentially-expressed in the microarray experiment.

### Validation of miRNA and mRNA differential expression

We selected a number of miRNA and mRNA for further analysis using qRTPCR. MiRNA candidates were selected based on novelty and mRNA targeting (miR-125a-5p, miR-145). The mRNA were chosen based on previously reported roles in drug addiction.

**Figure 3:**
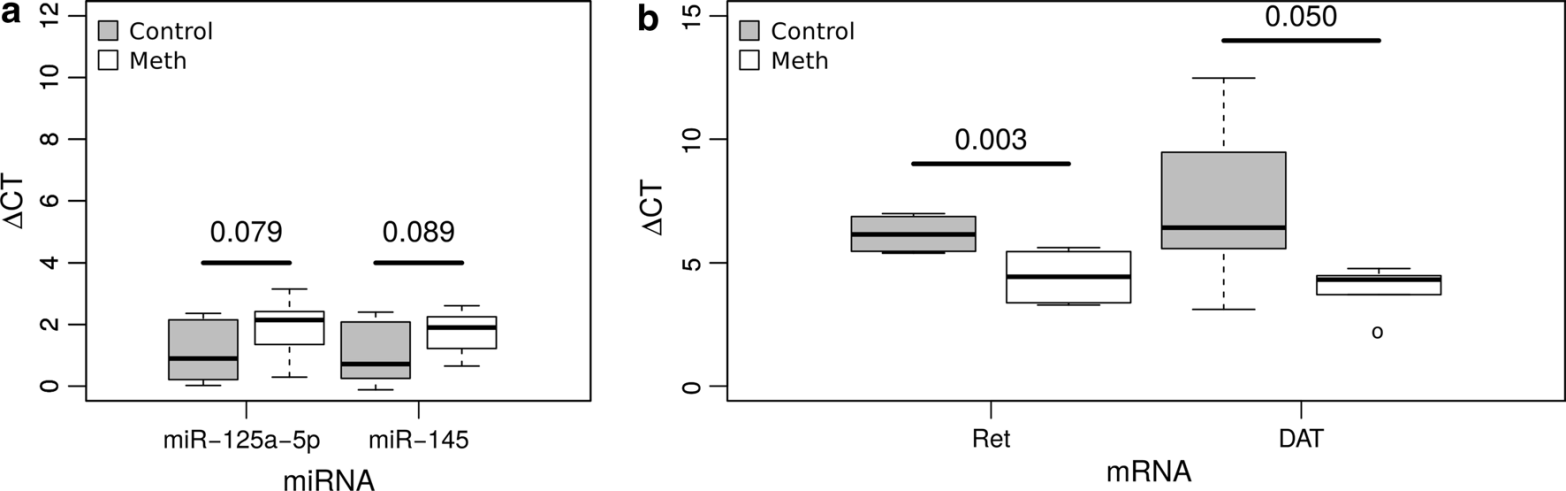
**a** qRTPCR expression levels for candidate miRNA. ∆Ct values (Ct_miRNA_ − Ct_U6_) are plotted on the Y axis. P-values for the difference between means (one-tailed T-test, as informed by the array data) are shown (n = 6 in each group). **b** qRTPCR expression levels for *DAT* and *Ret.* ∆Ct values (Ct_mRNA_ − Ct_GAPDH_) are plotted on the Y axis. There was a 3.3 fold-change upregulation of *Ret* and 10.8 fold-change upregulation of *DAT*. P-values for the difference between means (one-tailed T-test, as informed by the array data) are shown (control n = 5, methamphetamine n = 6).

In general agreement with the array data, we observed a trend towards downregulation of miR-125-a-5p (p = 0.079, fold change −1.79) and miR-145 (p = 0.089, fold change −1.82) in methamphetamine self-administration rats (Figure 3a, n = 6 in each group). In concordance with the array data, we observed a 3.3 fold-change significant upregulation of *Ret* (p < 0.01) and 10.8 fold-change significant upregulation of *DAT* (p < 0.05) mRNA in methamphetamine self-administration compared to drug naïve control rats (Figure 3b; n = 5 control, n = 6 methamphetamine).

## Discussion

We report the first combined mRNA and miRNA profiling of the VTA following methamphetamine self-administration and abstinence compared with drug naïve rats, using microarrays to identify significant changes in the level of 150 mRNA transcripts and 78 miRNAs. Methamphetamine is suggested to be either neurotoxic or neuroadaptive to neurons depending on the dose administered [25]. Our study sought to identify neuroadaptations associated with chronic low-dose methamphetamine by using a 2 h short-access model to identify persistent gene expression changes. The self-administration model is a well-established drug addiction model [26], provides greater face validity to human drug intake and provides a number of benefits for future gene expression studies; notably, gene expression patterns can be studied within the scope of behavioral correlates of human addiction, for example, reinstatement, relapse and escalation of intake. This study aimed to use self-administration to determine it’s suitability for the detection of long lasting gene expression changes in a small brain region. Long lasting gene expression changes in the VTA have been purported to be important in the addiction process [20, 27] and the effects of methamphetamine has not been studied in as much detail in the VTA as in the dorsal striatum and nucleus accumbens, areas of it’s primary pharmacologic effect. Our observations of differential mRNA expression suggest that the VTA plays an important role in response to long-term methamphetamine exposure, identifying many genes with known and potential roles in addiction.

We identified upregulation of mRNA expression for *DAT* and *Ret* (Table 1; Figure 3b), two genes with established roles in regulating dopamine levels in the VTA [20, 28]. DAT is the primary substrate of methamphetamine and inhibition or knockout of DAT prevents the pharmacological effects of methamphetamine (e.g. increased energy, euphoria). Methamphetamine can induce changes in DAT function and expression within the dorsal striatum and nucleus accumbens [29, 30] and thus affect drug-taking behavior. Unfortunately, protein samples were not available to us, but it will be important to determine whether these mRNA expression changes relate to differences at the protein level in future studies. In addition, our pathways analysis highlighted important dopaminergic cell markers such as TH and Nurr1 (Nr4a2), as well as a specific midbrain microRNA, miR-133b [31]. Further pathways analysis indicated enrichment for targets of two transcription factors, CREB and c-Myc, which both have identified roles in addiction (Figure 3a) [9, 32]. Increased phospho-CREB enrichment was reported on promoter regions of addiction-associated genes *c*-*fos*, *FosB*, *BDNF* and *synaptophysin* in the striatum following methamphetamine self-administration [13]. Synaptic activity leads to a prolonged phosphorylation of CREB, and multiple drugs of abuse lead to increased pCREB in the dStr and NAc, including cocaine [33, 34]. Our results suggest that CREB and c-Myc may have a role during abstinence, providing a possible mechanism for long term transcriptional changes following repeated drug exposure.

We observed differential mRNA expression of a number of other genes with known or putative roles in brain biology/addiction, but not previously reported for methamphetamine exposure: Slc47a2 which is a multi-drug and toxin extrusion transporter that removes organic cations and interacts with organic cation transporters [35]; Slc9a6 (NHE6) which has a role in neurological disease and axon and dendrite branching [36], and Netrin-1 which is involved in axon guidance and is important in both brain development and adult brain function. As the Netrin-1 receptor is upregulated in the VTA with repeated amphetamine exposure [37] it is possible that perturbations of netrin-1 may regulate vulnerability to relapse.

Pathways analysis of the 48/150 differentially expressed transcripts that were annotated revealed that proteins encoded by 18/48 were co-expressed or co-localized (Figure 2b), providing strong evidence for the potential relevance of this study to addiction biology. A number of these were cell surface receptors previously shown to be involved in drug addiction. The Tachykinin receptor (Tacr3, also known as neurokinin receptor Nk3R), has a role in reinforcement processes, and cocaine conditioned place preference can decrease methylation in the promoter region of this receptor [38]. Thus, future work investigating changes of methylation in the *Tacr3* promoter in response to methamphetamine is warranted. Single nucleotide polymorphisms in *Tacr3* have been associated with alcohol and cocaine dependence in humans [39], and thus genetic variation may also play a role in methamphetamine addiction. Nicotinic cholinergic receptor (Chrna6) is a subunit of nicotinic acetylcholine receptors expressed in the VTA and substantia nigra, has a role in both nicotine and alcohol administration [40] and influences dopamine levels in the dorsal striatum and NAc [41]. Thus, our observation of Chrna6 upregulation may be indicative of changes to dopaminergic systems following repeated methamphetamine exposure. In addition, heterogeneous nuclear ribonucleoprotein (Hnrnpa3) is expressed in the brain and has a role in mRNA maturation and Lin7a is a scaffold protein involved in neurite extension and filopodia formation in neurons [42]. Analyses of the 48 differentially expressed and annotated mRNA revealed genes with both known and previously unreported roles in addiction (as discussed above). Future analysis of the 102 unannotated transcripts may provide further biological insights.

The expression of miRNA is high in the central nervous system, which may indicate a particular importance for miRNA in this area of the body [15]. However, the study of miRNA within the brain following exposure to drugs of abuse is still a new field, with little known about the effects of drug exposure on global miRNA expression. We observed 78 miRNA with significant differential levels in the VTA between methamphetamine self-administration and drug naïve rats using microarray analyses. Strikingly, the majority of these (71) were downregulated on methamphetamine exposure and it is possible that methamphetamine exposure fundamentally alters the dynamics of miRNA expression in the VTA. Although speculation, this could be due to changes in methylation, chromatin remodeling or transcription factors. Given that the current model of miRNA regulation is transcriptional degradation or translational inhibition, it is possible that downregulation of miRNA removes a constitutive repression of genes that are important for conditions in the brain that maintain addiction-type behaviors. The increased expression of such genes may then be the important factor underlying the persistence of drug addiction even after long periods of abstinence. Further studies are required to investigate these possibilities and confirm changes in miRNA expression.

Upregulation of miR-181a following amphetamine exposure has been reported in the ventral midbrain [17], consistent with our observation of increased expression of the precursor transcript on methamphetamine self-administration. In the dorsal striatum, miR-212 influences cocaine addiction behaviors [43]. We observed a trend towards downregulation of miR-212 in the VTA on methamphetamine self-administration (Additional file 5: Figure S4). We were interested to observe the higher variability of miR-212 and mature miR-181a (from the miRNA array data) on methamphetamine self-administration compared to drug naïve controls (Additional file 5: Figure S4); this may be indicative of a dysregulation following methamphetamine exposure and warrants further investigation. In addition, we observed reduced expression of miR-9 and miR-140 expression consistent with that observed on ethanol exposure [44].

MiRNA bind to target region(s) in the 3′UTR of mRNA, and regulate mRNA expression via translational inhibition or transcriptional repression [19]. We identified mRNA with target sites in their 3′UTR for differentially-expressed miRNA and observed that 12/31 significantly upregulated mRNA contained such sites for miRNA downregulated in our parallel analyses (Table 1). For example, a significant decrease in miR-125a-5p was seen following methamphetamine administration along with increased *Ret* mRNA expression, one of its purported targets. miR-125a-5p has not previously been implicated in addiction. Pathways analysis of the 48 differentially-expressed and annotated mRNA showed enrichment for two miRNA which were significantly downregulated in the array analyses (Table 3). miR-9 has been linked to nicotine and ethanol exposure [44]; however, dysregulation of miR-145 has not previously been reported after administration of any drug of addiction. Validation experiments using qRTPCR showed a trend for miRNA-125a-5p and miR-145 in a consistent direction to the array data, however, this did not reach statistical significance. We suspect that this is due to the small sample size used for this complex self-administration experiment. Future experiments which knock-down specific miRNA of interest and investigate subsequent changes of the target mRNA and protein level will yield insights into the biological significance of our observations.

A number of the genes identified as part of the protein–protein interaction network (Figure 2b) are also potentially regulated by differentially-expressed miRNA in this study. For instance, we observed a decrease in the levels of 5 miRNA with predicted target sites in the *Foxa1* 3′UTR (miR-106b, miR-194, miR-30c, miR-30b-5p and miR-20a) along with increased *Foxa1* mRNA expression on methamphetamine exposure. Foxa1 is a member of the forkhead box family, is a marker for dopaminergic neurons, regulates dopamine neuron development in the midbrain [45], and is linked to the maintenance of the dopaminergic neuron phenotype [46]. Therefore, Foxa1 may represent a previously unrecognized mediator of methamphetamine effects, and further study of Foxa1 regulation of dopaminergic cells within the VTA as a modulator of persistent molecular changes in abstinence is warranted. A main feature of drug addiction is relapse months or years after last exposure to drug; therefore, alterations to transcription factors may be an important way for this addiction potential to be maintained.

Recent research implicates epigenetics as a mechanism for persistent gene expression changes due to repeated exposure to drugs of abuse [47]. We observed differential regulation of multiple transcripts related to epigenetic mechanisms and it is possible that these mechanisms hold the key to persistent changes. It is also possible that the general downregulation of miRNA we observed is modulated by these epigenetic mechanisms. DNA methylation, histone modification and miRNA all play a role in epigenetic regulation. Methamphetamine has previously been shown to increase mRNA expression of *DNA methyltransferase 1* (*Dnmt1*) [48]. Our study identified significant upregulation of a transcript annotated as “similar to DNA methyltransferase 3B (LOC690309)”, which overlaps the DNA methyltransferase 3B gene (*Dnmt3B*) and appears to contain an identical 3′UTR. In addition, 6 miRNA downregulated in our analyses (miR-26a, 29a/b/c, 222, and 383, Additional file 4: Table S1) are predicted to bind 3′UTR of *Dnmt3B*, with miR-222, miR-383 and miR-29b have demonstrated to directly affect *Dnmt3B* expression [49, 50]. We also identified differential expression of a number of miRNAs which target histone modification enzymes; miR-145 suppresses *histone deacetylase 2* (*HDAC2*) [51], miR-129 is predicted to target HDAC2 mRNA and miR-29 targets HDAC4 mRNA [52]. Long-non-coding RNA (lncRNA) are also involved in epigenetic regulation and their relevance to brain biology has recently been recognized [53]. On examination of the unannotated transcripts from the mRNA array against rat lncRNA databases [54] we identified one lncRNA (lincRNA7834551) with less expression in methamphetamine self-administration compared to drug-naïve controls (Additional file 6: Figure S5).

In human chronic users, methamphetamine administration occurs either in consistent low dose administration or cycles of high dose binges [55]. Users that are not classed as dependent may still take methamphetamine regularly for the purposes of alertness, concentration, increased energy or as a dieting aid. Despite the limitations of applying animal models to the human condition, we hypothesized that the model of self-administration we used would be potentially applicable to the human drug takers outlined above, and could thus be used to provide additional insights into this aspect of addiction biology. We believe that our identification of persistent expression changes in genes with both known and previously unknown roles in addiction and related biological pathways demonstrates the potential relevance and efficacy of this model in the study of addiction, providing a cost efficient model of drug taking. The study provides a preliminary insight into changes in the VTA following methamphetamine self-administration, representing drug-taking, rather than drug addiction. A number of key targets identified potentially provide a mechanistic insight into the effects of methamphetamine in the VTA and their functions can be further elucidated in experiments to relate them to the pharmacological effect of methamphetamine. Future work expanding this model to include longer access, non-contingent exposure, extinction and drug challenge should provide additional understanding reflective of human addiction.

## Conclusion

Our study demonstrates that short-access methamphetamine self-administration is a useful model to elucidate biologically meaningful changes in the brain. We observed a large number of changes in our microarray analyses to mRNA and miRNA levels with methamphetamine self-administration and found a strong relationship between addiction biology and the genes that were differentially-expressed, as well as clues towards the regulation of mRNA by miRNA. Our data suggests an important role for small RNA molecules in the regulation of gene expression changes in the VTA and that this may well influence vulnerability to addiction.

## Methods

### Animals

Male Sprague–Dawley rats (*Rattus norvegicus*, 300–350 g) were housed individually in hanging polycarbonate cages, at 19–21°C, and 55% humidity with 12 h light/dark cycling. Animals had ad libitum access to food and water except during self-administration training. All experiments were approved by and carried out in accordance with Animal Ethics Committee guidelines at Victoria University of Wellington. Animals were deeply anaesthetized (Ketamine, 90 mg/kg, I.P., Xylazine, 9 mg/kg, I.P.), fitted with chronic indwelling jugular catheters and assigned randomly to control (n = 11) or methamphetamine self-administration (n = 11) groups. Following 5 days of recovery post-surgery, rats received self-administration training for methamphetamine in standard operant chambers (Med Associates, ENV-001, St Albans, VT, USA) in the School of Psychology at Victoria University of Wellington using methods reported in previous studies [24], for review of the procedure, see [26]. Prior to each 2 h session, catheters were flushed with 0.2 mL heparin–penicillin solution. When the active lever was pressed, rats received a 12 s, 0.1 mL infusion of methamphetamine-HCl (BDG Synthesis, Wellington, NZ, USA, 0.1 mg/kg/infusion) dissolved in sterile heparinized (3 U/mL) physiological saline concurrent with illumination of a light above the active lever. Control animals received heparinized saline (3 U/mL) infusions upon depression of the active lever. Rats began on a fixed ratio-1 (FR-1) schedule of reinforcement, which gradually progressed to a FR-5 schedule using an intermediate FR-2 schedule, similar to published studies [56]. The requirements for progression between schedules was an active:inactive lever ratio of 2:1 and greater than 10 infusions per session for three consecutive days. On the FR-5 schedule, rats were run in daily sessions for 6 days/week. Rats were maintained on the FR-5 reinforcement schedule for 20 days prior to a 14 day forced abstinence period similar to previous work investigating gene expression changes in cocaine self-administering rats [27]. Lever responses were recorded using Med Associates software (MED-PC IV, version 4.2). Rats were euthanized by CO_2_ asphyxiation and decapitation. Brains were quickly removed and the VTA was rapidly dissected using an acrylic stereotaxic brain matrixes block (Alto, AgnTho’sAB, Sweden) on a glass Petri-dish on ice. The brain region coordinates (−6.72 mm from Bregma) were used according to the brain atlas of Paxinos and Watson [57] and all regions were freehand-dissected. Immediately following dissection, samples were homogenized in 400 μL Trizol^®^ (Life Technologies, Auckland, NZ, USA) and frozen at −80°C until use.

### RNA extraction

Total RNA was extracted using Trizol and a Zymo Direct-zol™ RNA MiniPrep kit (Ngaio Diagnostics, Nelson, NZ, USA) following the manufacturer’s protocol. Samples were eluted in 20 μL of RNase-free H_2_O, quantified using a Nanodrop ND-1000 (Thermo Fisher Scientific) spectrophotometer and RNA integrity (RIN) assessed using the Bioanalyzer 2100 (Agilent Technologies Inc. CA, USA). Samples with sufficient RNA to probe both array types (>230 ng total RNA) and a RIN >8 were accepted for microarray analysis. This resulted in seven control and seven methamphetamine samples to be used for array experiments.

### mRNA and miRNA gene expression arrays

Microarrays were carried out by New Zealand Genomics Limited (NZGL), at the University of Auckland facility. Total RNA (100 ng) was analyzed using the Affymetrix GeneChip Rat Exon 1.0 ST microarray. This chip has approximately 1 million probe sets, covering 850,000 exon clusters. 130 ng of total RNA was analyzed using the Affymetrix GeneChip microRNA 3.0 arrays which contain probes for 680 mature and 486 pre *Rattus norvegicus* miRNAs.

### Expression microarray analysis

Analyses were performed using R version 2.15.2 (http://www.r-project.org, RRID:nif-0000-10474) [58], Bioconductor packages (RRID:nif-0000-10445) [59] and custom bash scripts. The mRNA Affymetrix CEL files were imported into AROMA (http://www.aroma-project.org/, RRID:OMICS_00703) [60], an R package specifically developed for Affymetrix Exon arrays. mRNA data was background corrected, quantile normalized and log2 transformed prior to further analyses. The miRNA CEL files were analyzed using a combination of the affy (RRID:OMICS_00740) [61] and oligo [62] packages, and were also background corrected and log2 transformed prior to analysis of differential expression. Quality assessment after background correction revealed two samples (1 control and 1 methamphetamine self-administration) that were outliers; therefore these samples were removed from subsequent analyses. Normalized expression data were analyzed using the Limma R package (RRID:OMICS_00769) [63]. To account for some of the variance between arrays, the array weights function was used. All differential analyses included a correction for multiple testing using the Benjamini–Hochberg [BH] correction as implemented in R. Microarray data will be deposited in GEO.

### Pathways enrichment

Enrichment analyses were performed using WebGestalt (WEB-based Gene SeT AnaLysis Toolkit, http://bioinfo.vanderbilt.edu/WebGestalt, RRID:nif-0000-30622) [64]. Gene IDs were uploaded and analysis performed against the rat reference genome using Bonferroni adjusted threshold of p < 0.05 with a minimum observation of n = 2. GeneIDs for miRNA targets were obtained from miRBase (http://www.mirbase.org, RRID:nif-0000-03134). Enrichment analysis for transcription factors was performed using Metacore™ (Thomson Reuters, https://portal.genego.com, RRID:nif-0000-20874). In addition, we used GeneMANIA (http://www.genemania.org, RRID:nlx_149159) to generate a schematic overview of predicted protein–protein interactions of the 48 significantly differentially-expressed mRNA. These predictions are informed by evidence-based results, which include co-localization and co-expression. TargetScan (http://www.targetscan.org, RRID:OMICS_00420) was used to find predicted mRNA targets for miRNA identified as differentially-expressed.

### Quantitative real time polymerase chain reaction (qRTPCR)

Six control samples and six methamphetamine self-administration samples were used for qRTPCR validation, this included two from each group in the original array experiment.

### Reverse transcription PCR

miRNA: 50 ng total RNA was reverse transcribed to cDNA using the Taqman miRNA RT kit (#4366596), 2 mM dNTP, 100 U Multiscribe™ reverse transcriptase, 5 U RNase inhibitor, RT buffer and a pool of the five small RNA primers according to the manufacturer’s instructions (Applied Biosystems, Life Technologies). mRNA: 50 ng total RNA was reverse transcribed using the High capacity RNA-to-cDNA reverse transcription kit (Applied Biosystems, #4387406). All cDNA samples were stored at −20°C until use.

### Quantitative real-time PCR

miRNA qRTPCR analysis was performed using Taqman assays (Applied Biosystems) (Cat #4429795): miR-145 (Assay #2278), miR-125a-5p (Assay #2198). The endogenous control was U6 snRNA (Assay #1973). Analysis of mRNA expression for *Ret* and *DAT* used (Cat #4331182, Rn00562224_m1) and (Cat #4331182, Rn01463098_m1) respectively. GAPDH (Cat #4331182, Rn01775763_m1) was selected as an endogenous control [56]. Analyses were performed with a final volume of 10 µL of miRNA cDNA, or 20 µL of mRNA cDNA and Universal PCR Mastermix (#4369016) in a Bio-Rad CFX Connect Real-time system cycler (Bio-Rad, CA, USA). Each sample was run in triplicate. Expression was normalized (∆Ct) using the appropriate endogenous control; small nuclear RNA U6 for the miRNA, and GAPDH for the mRNA analyses. A one-tailed T-test (comparison of means) was used to test for significance, in line with the differential expression observed for the array data.

## Additional files

Additional file 1: Figure S1 Heatmap representing cluster analysis for the mRNA array data. Expression levels indicated from low (red) through to high (green). All probes plotted pass BH adjustment (p < 0.05). Additional file 2: Figure S2. Animation overlaying mRNA–mRNA data for intra-drug naïve and intra-methamphetamine self-administration correlations (Pearsons). Additional file 3: Figure S3. Heatmap representing cluster analysis for the miRNA array data. Expression levels indicated from low (red) through to high (yellow). All probes plotted pass BH adjustment (p < 0.05). Additional file 4: Table S1. List of 78 significantly differentially expressed miRNA with methamphetamine self-administration. Additional file 5: Figure S4. Boxplots showing expression levels of miR-181a and miR-212 on the arrays. A) Expression level of miR-181a precursor, miR-181a-2, on the mRNA Exon array, B) expression level of mature miR-181a transcript on the miRNA array, C) expression level of mature miR-212 transcript miR-212 on the miRNA array. Additional file 6: Figure S5. Expression of lincRNA7834551 on the mRNA Exon array.

## Abbreviations

miRNA: microRNA
NAc: nucleus accumbens
VTA: ventral tegmental area
