# Supplementary figures and images for: mRNA and microRNA analysis reveals modulation of biochemical pathways related to addiction in the ventral tegmental area of methamphetamine self-administering rats
Source: BMC Neurosci. 2015 Jul 19;16:43. doi: 10.1186/s12868-015-0186-y (PMC4506769; doi:10.1186/s12868-015-0186-y)

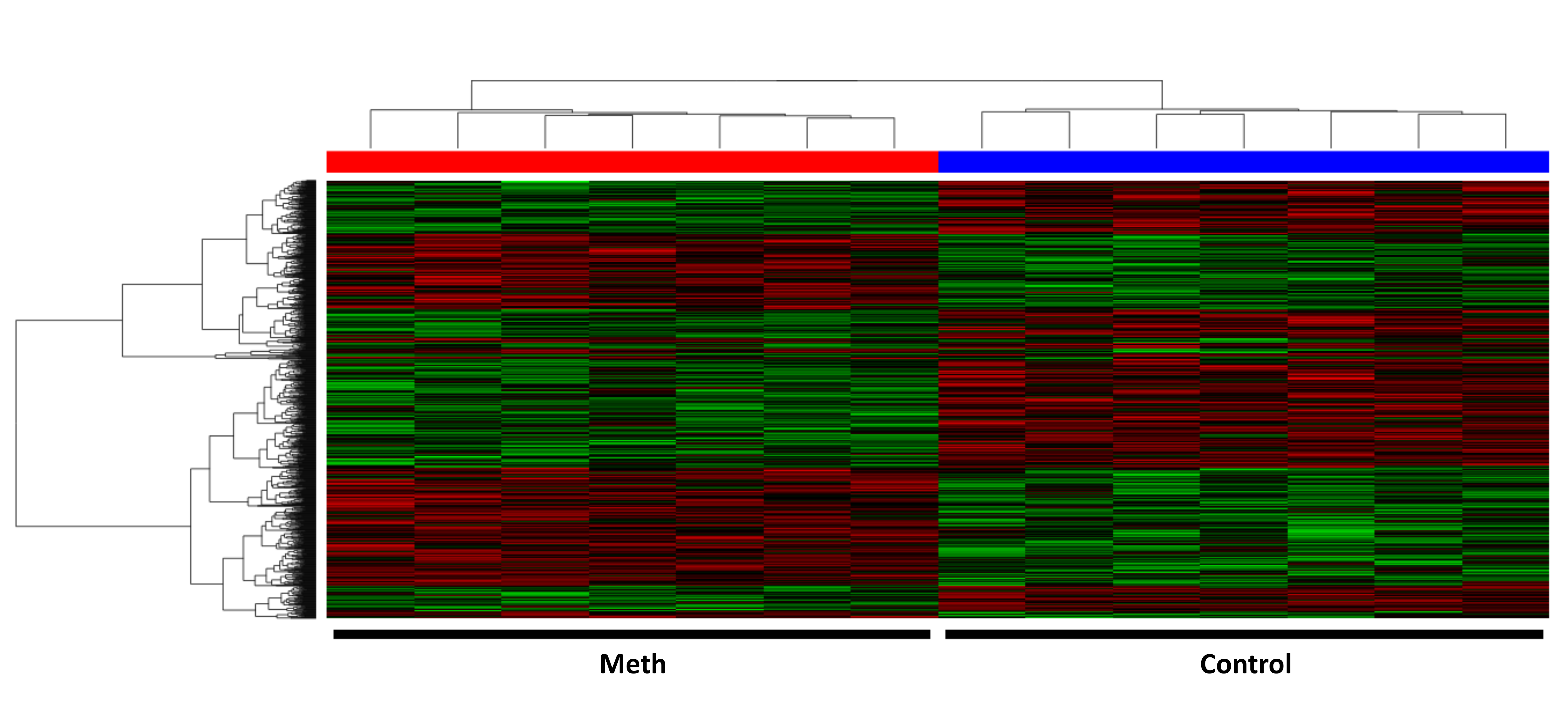

Supplement: Additional file 1: Figure S1 — Heatmap representing cluster analysis for the mRNA array data. Expression levels indicated from low (red) through to high (green). All probes plotted pass BH adjustment (p < 0.05). [file 12868_2015_186_MOESM1_ESM.tiff]

meth

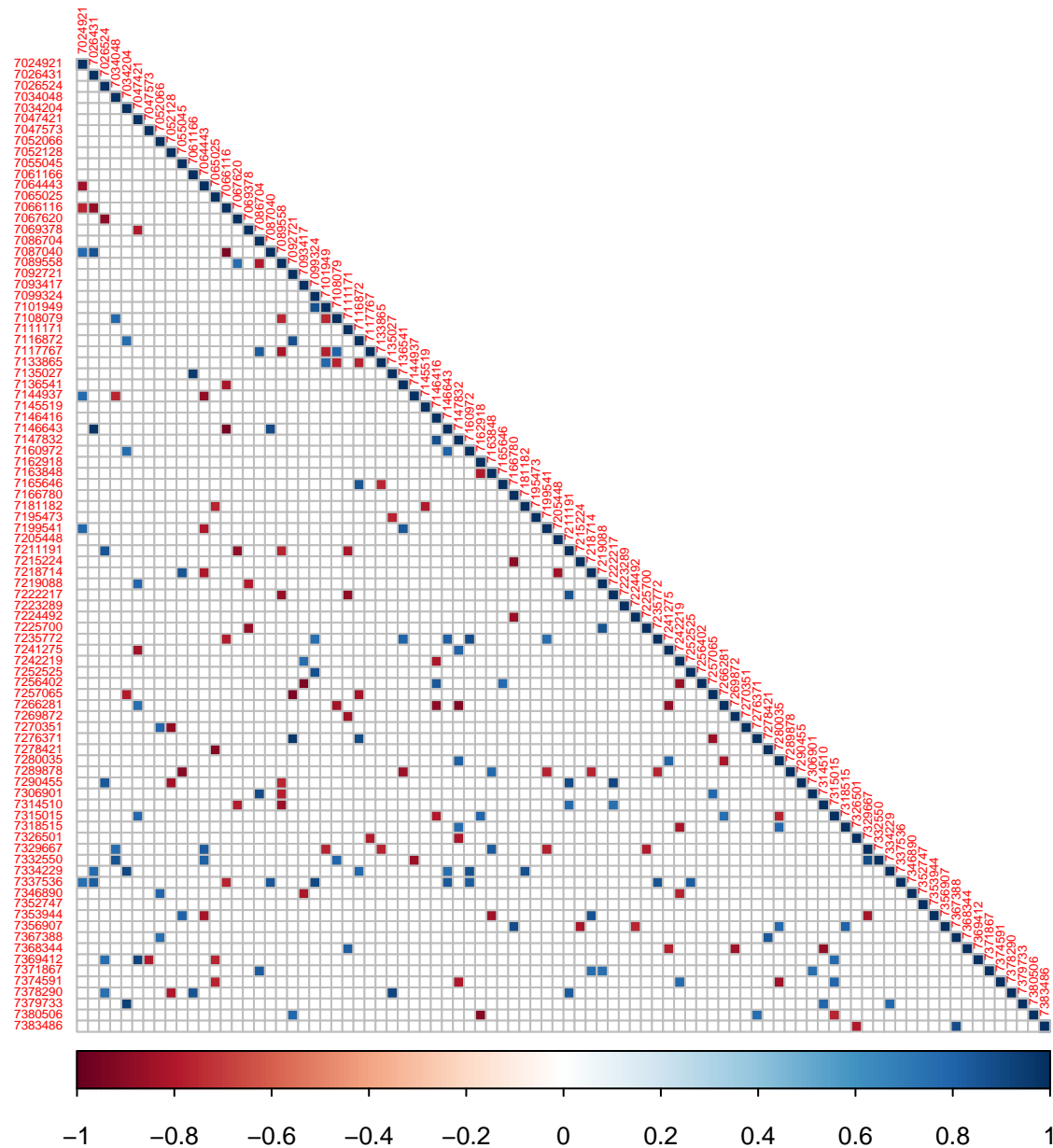

control

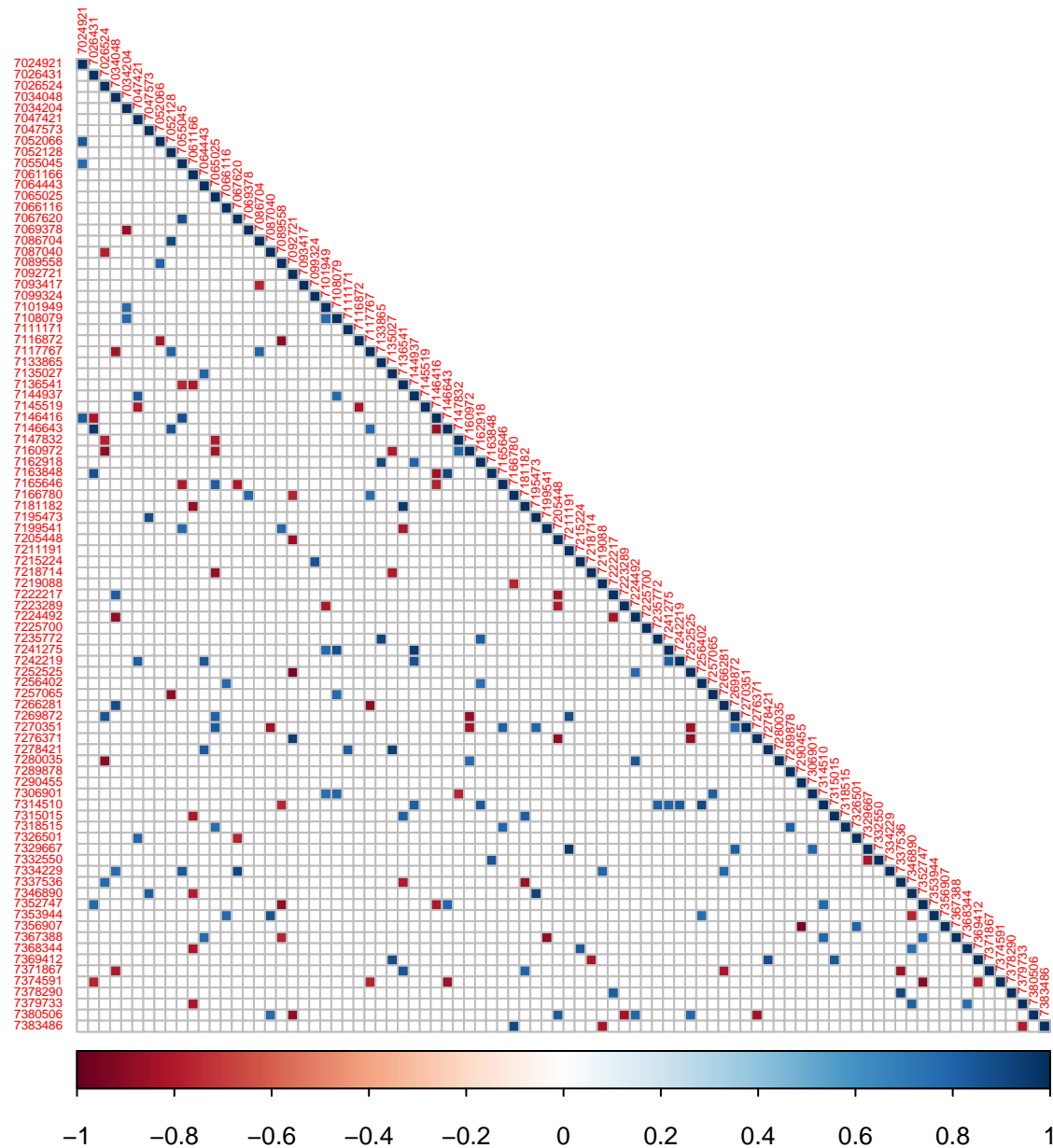

both

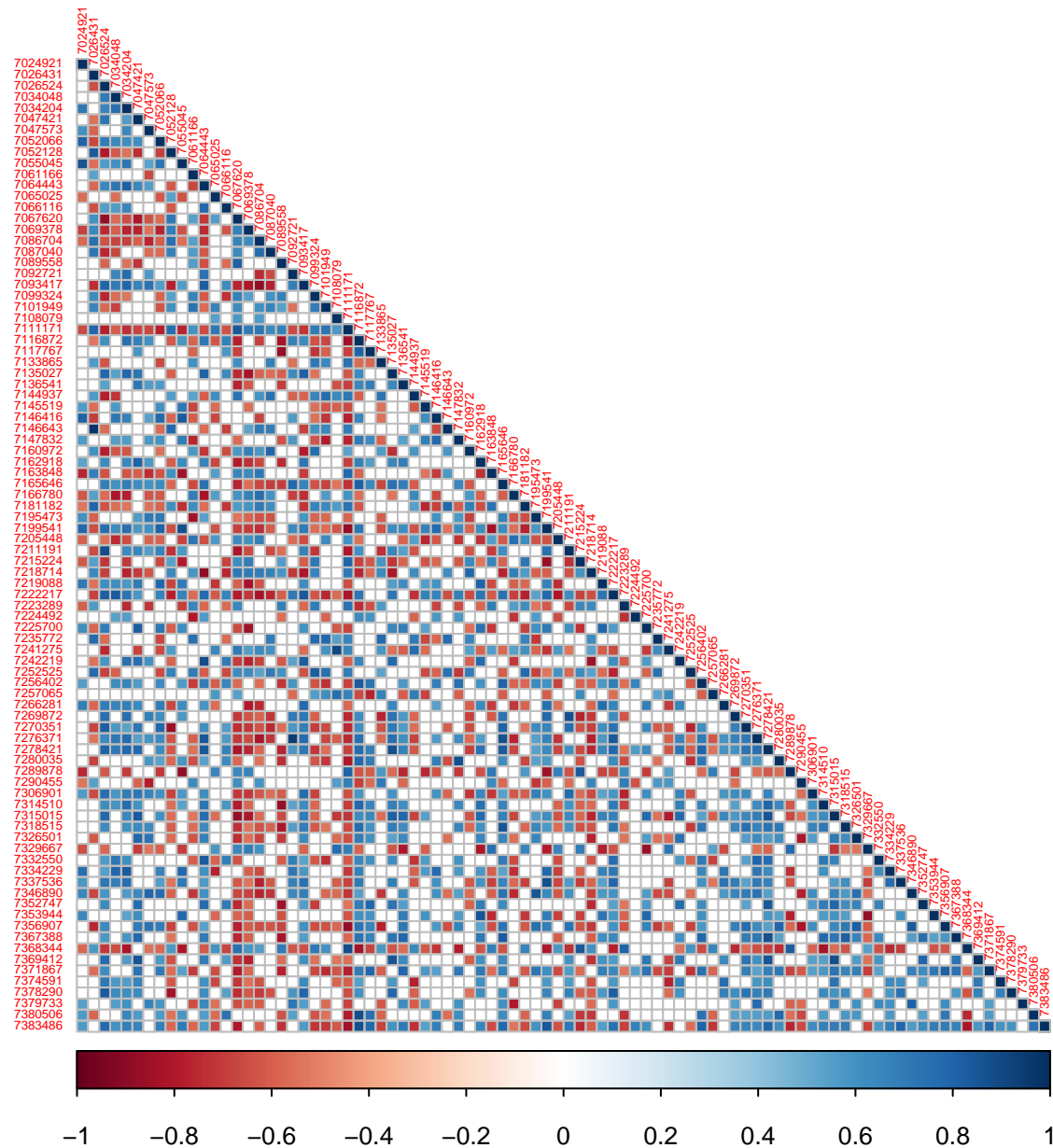

Supplement: Additional file 2: Figure S2. — Animation overlaying mRNA–mRNA data for intra-drug naïve and intra-methamphetamine self-administration correlations (Pearsons). [file 12868_2015_186_MOESM2_ESM.pdf]

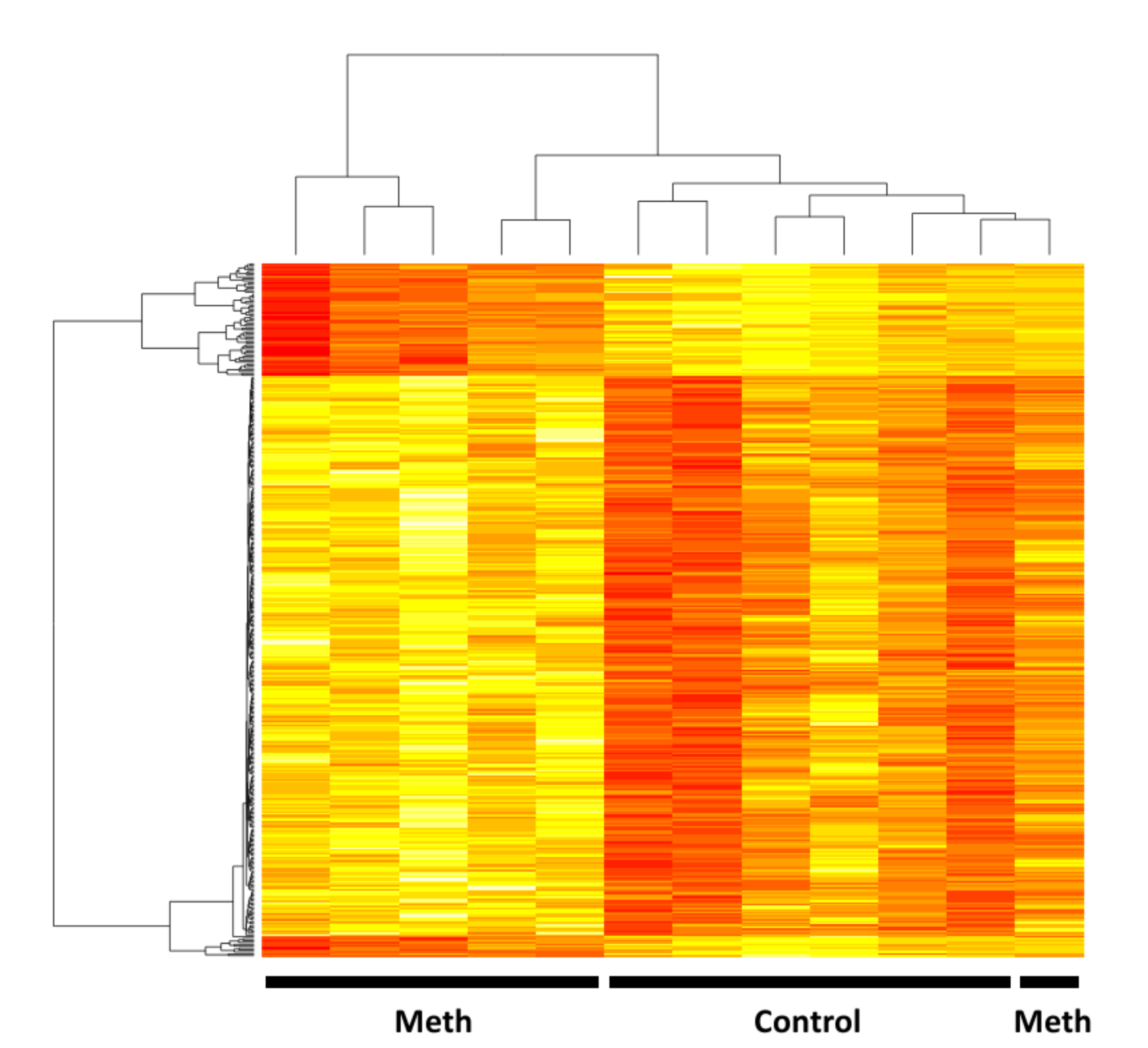

Supplement: Additional file 3: Figure S3. — Heatmap representing cluster analysis for the miRNA array data. Expression levels indicated from low (red) through to high (yellow). All probes plotted pass BH adjustment (p < 0.05). [file 12868_2015_186_MOESM3_ESM.tiff]

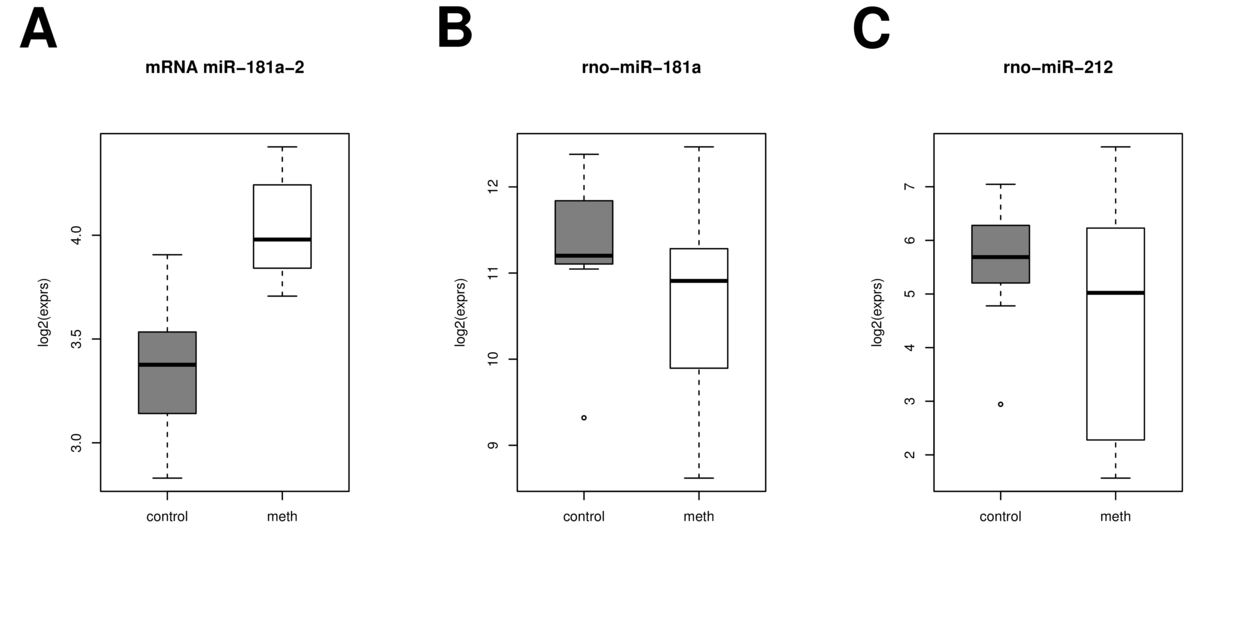

Supplement: Additional file 5: Figure S4. — Boxplots showing expression levels of miR-181a and miR-212 on the arrays. A) Expression level of miR-181a precursor, miR-181a-2, on the mRNA Exon array, B) expression level of mature miR-181a transcript on the miRNA array, C) expression level of mature miR-212 transcript miR-212 on the miRNA array. [file 12868_2015_186_MOESM5_ESM.tiff]

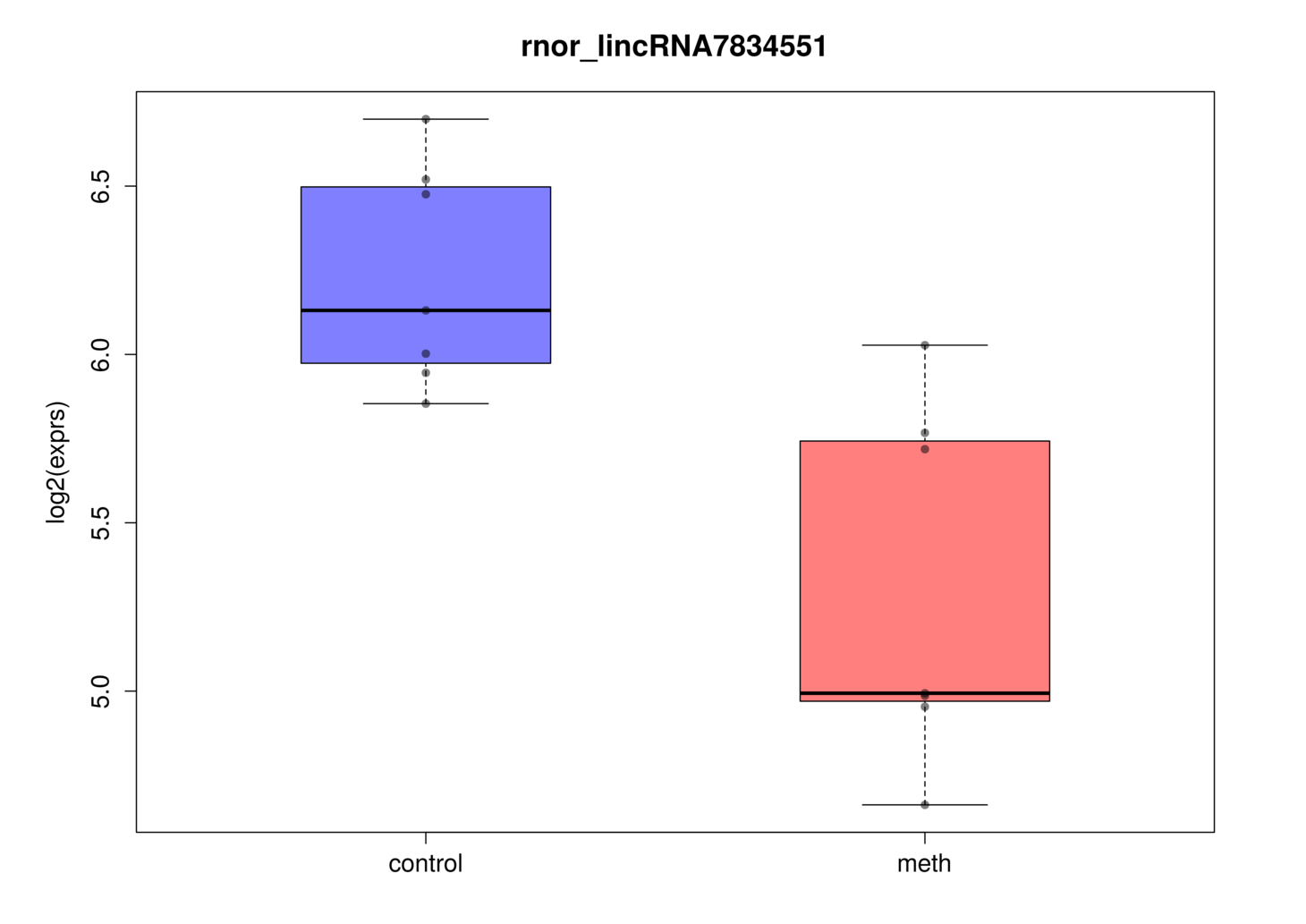

Supplement: Additional file 6: Figure S5. — Expression of lincRNA7834551 on the mRNA Exon array. [file 12868_2015_186_MOESM6_ESM.tiff]
