# Supplementary material for: mRNA and microRNA analysis reveals modulation of biochemical pathways related to addiction in the ventral tegmental area of methamphetamine self-administering rats
Source: BMC Neurosci. 2015 Jul 19;16:43. doi: 10.1186/s12868-015-0186-y (PMC4506769; doi:10.1186/s12868-015-0186-y)
Supplement: Additional file 4: Table S1. — List of 78 significantly differentially expressed miRNA with methamphetamine self-administration. [file 12868_2015_186_MOESM4_ESM.docx]

**Table S1: List of 78 significantly differentially expressed miRNA with methamphetamine self-administration**

| **Full I.D.** | **I.D.** | **Fold Change (log2)** | **Adj p-value** | **B** |
| --- | --- | --- | --- | --- |
| **Downregulated** |  |  |  |  |
| hp_rno-mir-351_st | Mir351 | 0.58 | 0.025 | 1.66 |
| rno-miR-129_st | Mir129 | 2.48 | 0.025 | 1.42 |
| rno-miR-29c_st | Mir29c | 2.39 | 0.025 | 1.30 |
| rno-miR-99a_st | Mir99a | 1.58 | 0.025 | 1.10 |
| rno-miR-99b_st | Mir99b | 1.10 | 0.025 | 1.03 |
| ***rno-miR-125a-5p_st*** | ***Mir125a-5p*** | ***1.25*** | ***0.025*** | ***0.66*** |
| rno-miR-191_st | Mir191 | 0.90 | 0.025 | 0.44 |
| rno-miR-30c_st | Mir30c | 1.39 | 0.025 | 0.18 |
| rno-miR-126_st | Mir126 | 1.76 | 0.025 | -0.05 |
| ***rno-miR-23a_st*** | ***Mir23a*** | ***1.28*** | ***0.025*** | ***-0.39*** |
| rno-miR-379_st | Mir379 | 1.25 | 0.025 | -0.46 |
| rno-miR-146b_st | Mir146b | 1.89 | 0.025 | -0.59 |
| rno-miR-29b-2*_st | Mir29b-2* | 2.28 | 0.025 | -0.66 |
| rno-miR-125b-5p_st | Mir125-b-5p | 0.95 | 0.025 | -0.69 |
| rno-miR-425_st | Mir425 | 1.13 | 0.026 | -0.80 |
| rno-miR-320_st | Mir320 | 0.91 | 0.026 | -0.85 |
| rno-miR-26a_st | Mir26a | 1.14 | 0.026 | -0.88 |
| rno-miR-146a_st | Mir146a | 2.35 | 0.026 | -0.92 |
| rno-miR-328a_st | Mir328a | 1.82 | 0.026 | -0.95 |
| rno-miR-24_st | Mir24 | 1.15 | 0.027 | -1.11 |
| rno-miR-383_st | Mir383 | 1.08 | 0.027 | -1.12 |
| rno-miR-22_st | Mir22 | 1.66 | 0.028 | -1.27 |
| rno-miR-361_st | Mir361 | 0.81 | 0.028 | -1.28 |
| rno-miR-23b_st | Mir23b | 0.87 | 0.028 | -1.31 |
| rno-miR-30b-5p_st | Mir30b-5p | 1.96 | 0.028 | -1.41 |
| rno-miR-30d_st | Mir30d | 2.31 | 0.028 | -1.43 |
| ***rno-miR-145_st*** | ***Mir145*** | ***1.49*** | ***0.028*** | ***-1.45*** |
| rno-miR-195_st | Mir195 | 1.59 | 0.028 | -1.45 |
| rno-miR-150_st | Mir150 | 1.14 | 0.028 | -1.46 |
| rno-miR-151_st | Mir151 | 1.17 | 0.029 | -1.49 |
| rno-miR-342-3p_st | Mir342-3p | 0.76 | 0.029 | -1.55 |
| rno-miR-16_st | Mir16 | 1.29 | 0.029 | -1.63 |
| rno-miR-130a_st | Mir130a | 1.69 | 0.029 | -1.64 |
| rno-let-7d_st | let7d | 0.82 | 0.031 | -1.86 |
| rno-miR-103_st | Mir103 | 1.04 | 0.031 | -1.87 |
| rno-miR-107_st | Mir107 | 1.09 | 0.031 | -1.91 |
| rno-miR-346_st | Mir346 | 0.89 | 0.031 | -1.92 |
| rno-miR-378_st | Mir378 | 2.50 | 0.031 | -1.94 |
| hp_rno-mir-124-3_s_st | Mir124-3 | 1.17 | 0.032 | -2.03 |
| rno-miR-100_st | Mir100 | 1.05 | 0.032 | -2.05 |
| rno-miR-382_st | Mir382 | 0.91 | 0.033 | -2.12 |
| rno-miR-185_st | Mir185 | 1.26 | 0.034 | -2.19 |
| rno-miR-129-2*_st | Mir129-2* | 1.29 | 0.034 | -2.21 |
| rno-let-7e_st | let7e | 0.67 | 0.034 | -2.22 |
| rno-miR-134_st | Mir134 | 1.09 | 0.034 | -2.22 |
| hp_rno-mir-124-1_s_st | Mir124-1 | 1.19 | 0.034 | -2.22 |
| hp_rno-mir-124-2_s_st | Mir124-2 | 1.15 | 0.034 | -2.23 |
| rno-miR-192_st | Mir192 | 2.32 | 0.035 | -2.31 |
| rno-miR-194_st | Mir194 | 2.13 | 0.035 | -2.31 |
| ***rno-miR-124_st*** | ***Mir124*** | ***1.27*** | ***0.035*** | ***-2.33*** |
| rno-miR-20a_st | Mir20a | 1.96 | 0.037 | -2.40 |
| rno-miR-143_st | Mir143 | 1.87 | 0.037 | -2.44 |
| rno-miR-411_st | Mir411 | 1.96 | 0.038 | -2.47 |
| rno-miR-140*_st | Mir140* | 1.50 | 0.038 | -2.48 |
| rno-miR-222_st | Mir222 | 1.65 | 0.039 | -2.52 |
| rno-miR-541_st | Mir541 | 0.87 | 0.040 | -2.56 |
| rno-miR-181d_st | Mir181d | 1.74 | 0.040 | -2.64 |
| rno-miR-128_st | Mir128 | 2.36 | 0.040 | -2.64 |
| rno-miR-127_st | Mir127 | 0.80 | 0.041 | -2.67 |
| rno-miR-29a_st | Mir29a | 1.91 | 0.041 | -2.67 |
| rno-miR-27a_st | Mir27a | 2.95 | 0.041 | -2.70 |
| rno-miR-345-5p_st | Mir345-5p | 1.05 | 0.041 | -2.70 |
| rno-miR-434_st | Mir434 | 1.05 | 0.041 | -2.72 |
| rno-miR-674-5p_st | Mir674-5p | 1.01 | 0.044 | -2.84 |
| rno-miR-30c-2*_st | Mir30c-2* | 1.30 | 0.044 | -2.84 |
| rno-miR-221_st | Mir221 | 1.94 | 0.045 | -2.86 |
| rno-miR-125b-3p_st | Mir125b-3p | 1.09 | 0.045 | -2.86 |
| rno-miR-652_st | Mir652 | 1.41 | 0.047 | -2.92 |
| rno-miR-27b_st | Mir27b | 1.66 | 0.049 | -3.01 |
| rno-miR-9*_st | Mir9* | 2.36 | 0.049 | -3.03 |
| rno-miR-106b_st | Mir106b | 2.31 | 0.049 | -3.06 |
| **Upregulated** |  |  |  |  |
| rno-miR-741-3p_st | Mir741-3p | -0.51 | 0.025 | 1.26 |
| rno-miR-3570_st | Mir3570 | -0.53 | 0.025 | -0.55 |
| rno-miR-369-3p_st | Mir369-3p | -0.60 | 0.025 | -0.52 |
| rno-miR-145*_st | Mir145* | -0.50 | 0.029 | -1.81 |
| hp_rno-mir-216b_st | Mir216b | -0.60 | 0.033 | -2.09 |
| hp_rno-mir-17-1_st | Mir17-1 | -0.57 | 0.032 | -2.03 |
| hp_rno-mir-181b-1_st | Mir181b-1 | -0.54 | 0.033 | -2.13 |

**Note:** miRNA in bold italics were selected for qRTPCR validation (see Methods)
